# Predicting online information seeking on Douyin, Baidu, and other Chinese search engines among gynecologic oncology patients: a cross-sectional study

**DOI:** 10.3389/fpsyg.2023.1255604

**Published:** 2023-11-23

**Authors:** Wenli Xiong, Xiaohong Li, Yun Han, Lijuan He

**Affiliations:** ^1^Health Management Center, Affiliated Hospital of Southwest Medical University, Luzhou, Sichuan, China; ^2^Department of Urology, Yibin Fifth People’s Hospital, Yibin, Sichuan, China

**Keywords:** gynecologic oncology, information seeking, online platforms, Douyin, Baidu, anxiety, depression, prediction model

## Abstract

**Objective:**

The rise of online platforms like Douyin, Baidu, and other Chinese search engines has changed how gynecologic oncology patients seek information about their diagnosis or condition. This study aimed to investigate the factors associated with information seeking among these patients and to evaluate their predictive performance.

**Methods:**

A cross-sectional study was conducted among 199 gynecologic oncology patients at a single hospital in China. The patients’ demographic characteristics and scores on the State–Trait Anxiety Inventory (STAI-S and STAI-T) and the Hospital Anxiety and Depression Scale (HADS-A and HADS-D) were compared between those who sought information online and those who did not. Logistic regression analyses and receiver operating characteristic (ROC) curve analyses were performed.

**Results:**

The patients’ age, marital status, STAI-S scores, and HADS-A scores were significantly associated with online information seeking. The combined model that included these factors showed good predictive performance with an area under the ROC curve of 0.841.

**Conclusion:**

The combination of demographic and psychological factors can be used to predict the likelihood of gynecologic oncology patients seeking information online. These findings can help healthcare providers understand their patients’ information-seeking behaviors and tailor their communication strategies accordingly.

## Introduction

In the digital age, the internet plays an increasingly central role in the way patients seek and obtain health-related information. Online platforms have grown to become a vital source of health information for patients, enabling them to learn about their conditions, understand treatment options, and engage more effectively in their healthcare (Moorhead et al., 2013; Duggan et al., 2014; Amante et al., 2015). In China, the trend of online health information seeking is pronounced, with individuals frequently turning to the internet for health science popularization, health behaviors, traditional Chinese medicine, and medical inquiries (Xiong et al., 2021). The outbreak of Severe Acute Respiratory Syndrome (SARS) in 2003 marked a significant rise in online health information-seeking behaviors, leading to the burgeoning of health websites in the country (Cao et al., 2016).

With over 989 million active users, Douyin, known internationally as TikTok, has emerged as one of China’s most popular social media platforms (Iqbal, 2021). In addition, Baidu, China’s leading search engine, attracts hundreds of millions of users each month (McDougall, 2014). These platforms and other Chinese search engines have significantly altered the landscape of patient education and health information seeking. A particular study among college students in Guangdong Province revealed that individuals sought health information primarily for self-care, general health, disease prevention, self-medication, family treatment, and drug information, among others, utilizing platforms like Baike.baidu.com, Zhihu.com, and Zhidao.baidu.com (Zhang et al., 2021).

A key demographic group navigating this digital landscape is patients diagnosed with gynecologic cancers, a group of cancers that affect women’s reproductive organs. These include cervical cancer, ovarian cancer, uterine cancer, vaginal cancer, and vulvar cancer, which together represent a significant health burden for women worldwide (Bray et al., 2018). As the complexity of gynecologic oncology increases with advancements in diagnosis and treatment modalities, so too does the necessity for patients to seek comprehensible and reliable information about their condition (Miller et al., 2022). This narrative aligns with the overarching themes elucidated in a comprehensive review on gynecologic oncology, emphasizing the paramountcy of accurate diagnostic methodologies and prognostic stratifications in enhancing patients’ survival and quality of life amidst the battle against gynecological malignancies (Perelli et al., 2023).

The process of seeking health information online is influenced by a myriad of factors, including demographic characteristics such as age, gender, education level, and income, as well as psychological factors like anxiety and depression (Gray et al., 2005; Wang et al., 2013; Tennant et al., 2015). The State–Trait Anxiety Inventory (STAI) and the Hospital Anxiety and Depression Scale (HADS) are among the validated instruments used to measure these psychological states (Spielberger et al., 1971; Zigmond and Snaith, 1983). However, the specific factors associated with online information seeking among gynecologic oncology patients, particularly on platforms like Douyin and Baidu, remain poorly understood.

Given these online platforms’ growing prevalence and potential impact on patient health outcomes, a comprehensive understanding of the characteristics and psychological states influencing a patient’s propensity to seek online health information is critical. Studies have demonstrated that patients who actively seek health information tend to be more involved in decision-making about their health, exhibit better self-management of their conditions, and generally have better health outcomes (McMullan, 2006; Lambert and Loiselle, 2007). However, it is also worth noting that the information seeking process can result in increased anxiety and distress, especially if the information obtained is conflicting, misunderstood, or if the patient stumbles upon worst-case scenarios (Broom, 2005; Leykin et al., 2012). Thus, it is crucial to identify which patients are more likely to seek online health information and understand their psychological states to provide appropriate guidance and support.

Further, in the era of personalized medicine, it is crucial to use predictive models that allow healthcare professionals to identify patients who are more likely to seek online information about their diagnosis or condition. These predictive models can aid in tailoring patient education and communication strategies, thus enhancing patient care (Schapira et al., 2017). Although numerous studies have focused on the relationship between internet use and health outcomes, few have attempted to develop predictive models for online information seeking behavior (Leung and Lee, 2005; Lee et al., 2014). The vast landscape of online health information in China and the particular preferences of individuals in seeking such information underscore the importance of our case study.

Our study seeks to fill the gap in understanding the online health information-seeking behaviors among gynecologic oncology patients in China, leveraging demographic and psychological data. By doing so, we aim to build a predictive model that could help clinicians better understand their patients’ information-seeking behaviors, inform strategies for patient communication and education, and contribute to improved patient outcomes in the realm of gynecologic oncology.

## Methods

### Study design and participants

This was a cross-sectional study conducted among gynecologic oncology patients at a single hospital in China utilizing a convenience sampling method. All patients visiting the Gynecologic Oncology department during the study period and meeting the inclusion criteria were invited to participate. The study included patients who were over 18 years of age, had a confirmed diagnosis of gynecologic cancer, and had the ability to use online platforms such as Douyin, Baidu, and other Chinese search engines. Patients were excluded if they were unable to comprehend or complete the survey. The types of gynecological cancers considered in this study included Ovarian Cancer, Uterine Cancer, and Cervical Cancer. In total, 199 patients participated in the study, 91 of whom sought information about their diagnosis or condition online, and 108 of whom did not.

### Data collection

A structured questionnaire was administered through face-to-face interviews conducted by trained medical staff. Data was collected on patients’ demographic characteristics, including age, marital status, education level, and annual income. The patients’ psychological states were assessed using the State–Trait Anxiety Inventory (STAI-S and STAI-T) and the Hospital Anxiety and Depression Scale (HADS-A and HADS-D). Participants were assured of the confidentiality of their responses, with data being anonymized before analysis and securely stored with restricted access.

### Translation, validation, and administration of questionnaires

The State–Trait Anxiety Inventory (STAI) and the Hospital Anxiety and Depression Scale (HADS) were translated into Mandarin Chinese by bilingual experts, using a forward and backward translation method to ensure accuracy. A small sample of gynecologic oncology patients participated in cognitive interviews to assess the clarity and relevance of the translated questionnaires. Previous studies have validated the translated versions of STAI and HADS in Mandarin Chinese, showing satisfactory psychometric properties. Our preliminary analysis also confirmed good internal consistency with Cronbach’s alpha values consistent with those reported in existing literature. Questionnaires were administered by trained research staff fluent in Mandarin Chinese, with assistance provided to participants as needed to clarify any uncertainties and ensure accurate responses.

### Statistical analysis

Descriptive statistics were used to summarize the characteristics of the patients. Chi-square tests and t-tests were conducted to compare the demographic and psychological characteristics between patients who sought information online and those who did not. Univariate and multivariate logistic regression analyses were used to identify the factors associated with online information seeking. The performance of the identified predictors was assessed using receiver operating characteristic (ROC) curve analysis, and the optimal cut-off values were determined based on the Youden index. All statistical analyses were conducted using R software, with a *p*-value of less than 0.05 considered statistically significant.

## Results

### Characteristics of gynecologic oncology patients seeking information on Douyin, Baidu, and other Chinese search engines

Table 1 provides an overview of the demographic and psychological characteristics of the gynecologic oncology patients in our study who used platforms such as Douyin, Baidu, and other Chinese search engines to seek information about their diagnosis or condition, compared to those who did not. The table shows the data for both groups, including the number of patients (91 who used the platforms and 108 who did not), average age, marital status, educational background, annual income, as well as scores on the STAI-S, STAI-T, HADS-A, and HADS-D psychological scales. From this table, we can see some statistically significant differences between the two groups. Patients who sought information on these platforms were younger on average (54.956 ± 8.029 years) compared to those who did not (62.593 ± 6.6206 years, *p* < 0.001). Marital status also varied significantly between the groups (*p* = 0.045), with a larger proportion of unmarried patients in the group that did not use these platforms. Differences in education levels were striking, with a significantly larger percentage of patients with high school education or less in the group not using the platforms. Annual income also varied, with the group using the platforms having a larger percentage in the higher income brackets. Psychologically, patients who sought information had higher STAI-S and STAI-T scores, indicative of greater levels of state and trait anxiety, respectively. Their median HADS-A scores were also higher, suggesting more symptoms of anxiety, but there was no significant difference in the HADS-D scores, indicating depressive symptoms.

**Table 1:** Characteristics of gynecologic oncology patients seeking information on Douyin, Baidu, and other Chinese search engines.

Characteristics	Yes	No	*p* value
*n*	91	108	
Age, mean ± sd	54.956 ± 8.029	62.593 ± 6.6206	<0.001
marital_status, *n* (%)			0.045
Unmarried	31 (34%)	52 (48.1%)	
Married/partnered	60 (66%)	56 (51.9%)	
Education, *n* (%)			<0.001
<High school	2 (2.2%)	8 (7.4%)	
High school	17 (18.7%)	30 (27.8%)	
Technical school/some college	18 (19.8%)	39 (36.1%)	
College degree (3- or 4-year)	35 (38.5%)	20 (18.5%)	
Graduate/professional degree	19 (20.9%)	11 (10.2%)	
annual_income, *n* (%)			0.010
<25,000	11 (12.1%)	28 (25.9%)	
25,000-49,000	17 (18.7%)	17 (15.7%)	
50,000-99,000	31 (34.1%)	24 (22.2%)	
≥100,000	29 (31.9%)	26 (24.1%)	
Not reported	3 (3.3%)	13 (12%)	
stai_s, mean ± sd	38.352 ± 5.7684	34.759 ± 5.9811	< 0.001
stai_t, mean ± sd	36.308 ± 5.4154	34.731 ± 5.4578	0.043
hads_a, median (IQR)	7 (6, 9)	6 (4.75, 8)	< 0.001
hads_d, median (IQR)	4 (3, 6)	4 (2, 5.25)	0.139

The table categorizes patients based on their online information-seeking behavior (Yes or No). Age is shown as mean ± standard deviation (sd). Marital status, education, and annual income are given as number (*n*) and percentage (%). State–Trait Anxiety Inventory (STAI) scores are denoted as state anxiety (stai_s) and trait anxiety (stai_t), both shown as mean ± sd. Hospital Anxiety and Depression Scale (HADS) scores are split into anxiety (hads_a) and depression (hads_d), and expressed as median with interquartile range (IQR). *p* values less than 0.05 indicate statistical significance.

### Univariate and multivariate analysis of factors associated with information seeking on Douyin, Baidu, and other Chinese search engines among gynecologic oncology patients

Table 2 elucidates the outcomes of both univariate and multivariate analyses probing the factors associated with gynecologic oncology patients seeking information regarding their diagnosis or condition on platforms like Douyin, Baidu, and other Chinese search engines. The table delineates odds ratios, confidence intervals, and *p*-values for each scrutinized factor, encompassing age, marital status, and scores on the STAI-S, STAI-T, HADS-A, and HADS-D psychological scales. In the univariate analysis, age exhibited a significant association with information-seeking behavior, presenting an odds ratio of 0.870 (95% CI: 0.827–0.915, *p* < 0.001). This association was further accentuated in the multivariate analysis, with an odds ratio of 0.853 (95% CI: 0.807–0.901, p < 0.001), indicating a decrease in the likelihood of seeking information online with advancing age. When examining marital status, the ‘married/partnered’ category was juxtaposed against the ‘unmarried’ category, serving as the reference group. Both univariate and multivariate analyses disclosed that being married or partnered was linked with higher odds of seeking information (OR = 2.25, 95% CI: 1.25–4.05, *p* = 0.008 in univariate; OR = 2.50, 95% CI: 1.40–4.45, *p* = 0.002 in multivariate). Additionally, scores on the STAI-S, STAI-T, and HADS-A scales were significantly associated with information seeking in both univariate and multivariate analyses, suggesting a relationship between certain psychological well-being facets and the propensity to seek information online. Conversely, HADS-D scores, indicative of depressive symptoms, demonstrated no significant association in the univariate analysis and hence, were excluded from the multivariate analysis.

**Table 2:** Univariate and multivariate analysis of factors associated with information seeking on Douyin, Baidu, and other Chinese search engines among gynecologic oncology patients.

Characteristics	Total (*N*)	Univariate analysis		Multivariate analysis	
		Odds Ratio (95% CI)	*p* value	Odds Ratio (95% CI)	*p* value
Age	199	0.870 (0.827–0.915)	**<0.001**	0.853 (0.807–0.901)	**<0.001**
marital_status	199				
Unmarried	83	Reference		Reference	
Married/partnered	116	2.25 (1.25–4.05)	**0.008**	2.50 (1.40–4.45)	**0.002**
stai_s	199	1.102 (1.050–1.157)	**<0.001**	1.095 (1.042–1.151)	**<0.001**
stai_t	199	1.080 (1.020–1.143)	**0.039**	1.073 (1.013–1.138)	0.216
hads_a	199	1.150 (1.080–1.225)	**<0.001**	1.130 (1.060–1.205)	**<0.001**
hads_d	199	1.000 (0.900–1.100)	0.139		

The table presents a univariate and multivariate analysis of different characteristics and their association with online information-seeking behavior among the participants. The total number of participants (*N*) is 199. Odds Ratios (OR) with 95% Confidence Intervals (95% CI) and *p* values are provided for both univariate and multivariate analysis. The Reference category in marital_status represents the baseline group for comparison. *p* values less than 0.05 are considered statistically significant.

### Performance of age, STAI-S, STAI-T, HADS-A, and the combined model in predicting information seeking on Douyin, Baidu, and other Chinese search engines among gynecologic oncology patients

**Figure 1:**
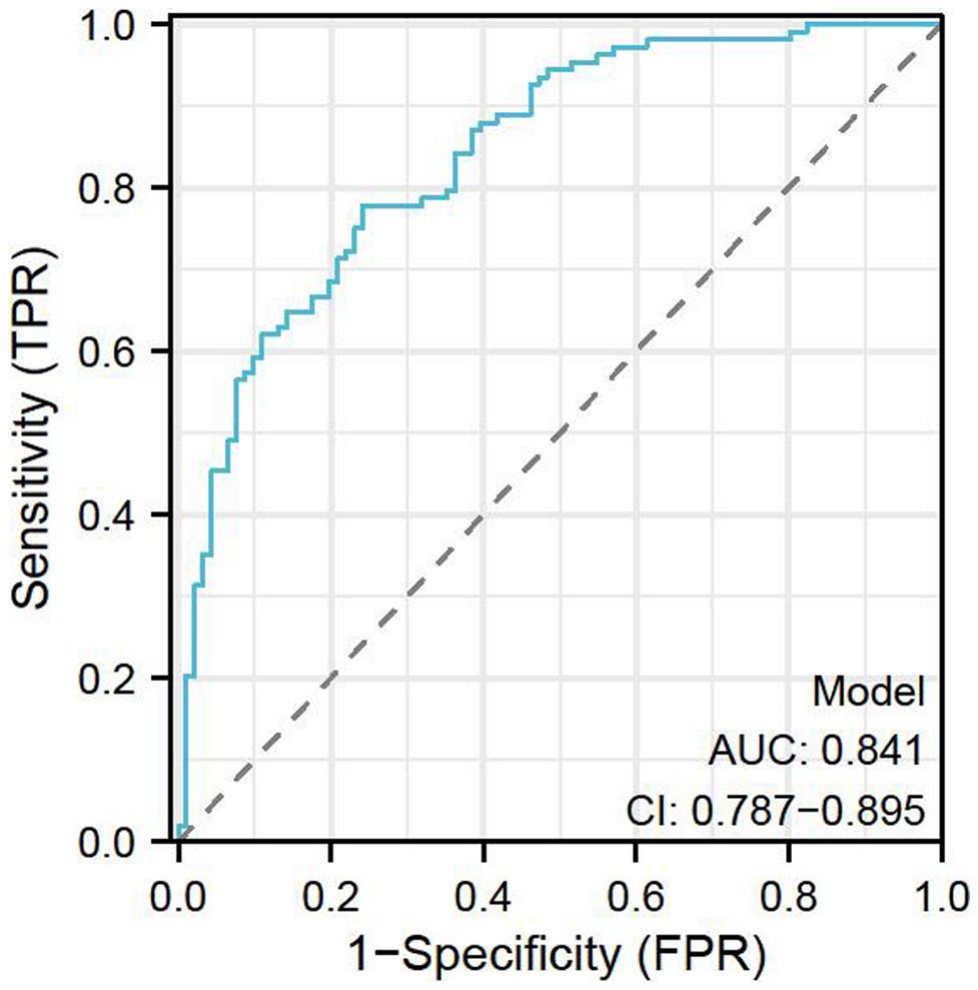
A ROC curve for the model.

Table 3 compares the performance of age, STAI-S, STAI-T, and HADS-A scores, as well as a combined model, in predicting whether gynecologic oncology patients would seek information about their diagnosis or condition on platforms like Douyin, Baidu, and other Chinese search engines. The parameters evaluated include area under the receiver operating characteristic curve (AUC), 95% confidence interval (CI), cut-off values for each predictor, and specificity and sensitivity at these cut-off values. For each of the predictors, age performed the best with an AUC of 0.776 (95% CI: 0.709–0.843), a cut-off value of 57.5, specificity of 64.835%, and sensitivity of 81.481%. The STAI-S and HADS-A scores showed moderate performance, with AUCs of 0.673 (95% CI: 0.598–0.747) and 0.645 (95% CI: 0.569–0.721), respectively. The STAI-T scores had the lowest AUC at 0.591 (95% CI: 0.511–0.670). The combined model, which takes all these factors into account, outperformed any single predictor, with an AUC of 0.841 (95% CI: 0.787–0.895), specificity of 75.824%, and sensitivity of 77.778%. These results highlight the potential of using a combination of demographic and psychological factors to predict information-seeking behavior in this patient population. A ROC curve was plotted for the model, see Figure 1.

**Table 3:** Performance of age, STAI-S, STAI-T, HADS-A, and the combined model in predicting information seeking on Douyin, Baidu, and other Chinese search engines among gynecologic oncology patients.

Parameters	AUC	95% CI	Cut-off value	Specificity (%)	Sensitivity (%)
Age	0.776	0.709–0.843	57.5	0.64835	0.81481
stai_s	0.673	0.598–0.747	36.5	0.61538	0.65741
stai_t	0.591	0.511–0.670	37.5	0.40659	0.75926
hads_a	0.645	0.569–0.721	5.5	0.8022	0.41667
Model	0.841	0.787–0.895	0.14178	0.75824	0.77778

This table presents the performance of different parameters and a combined model in predicting the information-seeking behavior on Douyin, Baidu, and other Chinese search engines among the participants. The parameters include age, state anxiety (STAI-S), trait anxiety (STAI-T), and anxiety from the Hospital Anxiety and Depression Scale (HADS-A). The Area Under the Curve (AUC) with 95% Confidence Intervals (95% CI) are provided to show the performance of each parameter and the combined model in predicting information-seeking behavior. The cut-off values, specificity, and sensitivity percentages are also provided for each parameter and the combined model to further evaluate their predictive accuracy.

## Discussion

This research illuminates the online health information-seeking behaviors among gynecologic oncology patients in China, a demographic navigating the digital landscape primarily through popular platforms like Douyin and Baidu. The comprehensive analysis conducted in this study underscores the critical influence of demographic and psychological factors on these behaviors.

China, with its vast population and rapidly evolving digital infrastructure, presents a unique context for exploring online health information-seeking behaviors. The penetration of internet services and digital platforms has significantly increased over the years, enabling a large segment of the population to access online resources for health-related information. This backdrop provides a fertile ground for understanding the interplay of demographic and psychological factors in influencing online health information-seeking tendencies among gynecologic oncology patients.

The association of older age with reduced online information seeking, as observed in our study, aligns with previous studies (Smith, 2014), hinting at a digital divide based on age. However, ongoing digital literacy initiatives and the proliferation of internet-enabled devices may potentially bridge this divide over time, a trajectory worthy of investigation in future research (Anderson and Perrin, 2017).

Furthermore, the differing online information-seeking behaviors between married or partnered individuals and their unmarried counterparts open avenues for exploring the role of social support in health information-seeking processes (LaCoursiere et al., 2005). The intricacies of social dynamics and their impact on online health information-seeking behaviors could be a focal point for future research.

From a psychological lens, our findings accentuate the role of state anxiety in online health information-seeking behavior. Although previous research has highlighted the roles of both anxiety and depression in health information seeking (Eastin and Guinsler, 2006; Bessière et al., 2010), our study uniquely reveals that only state anxiety significantly associates with this behavior within the Chinese context, where mental health symptoms may manifest or be reported differently (Hsiao et al., 2006).

The predictive model crafted in this study serves as a robust tool in forecasting online health information-seeking behaviors among gynecologic oncology patients, holding substantial implications for healthcare providers. By understanding a patient’s demographic and psychological profile, healthcare providers can tailor communication strategies to better meet the informational needs of their patients.

Nonetheless, the model’s predictive prowess was not flawless, hinting at other possibly influential factors not encapsulated in our study. Future explorations could delve into additional predictors like health literacy, perceived health status, and trust in online health information to further refine the understanding of online health information-seeking behaviors among gynecologic oncology patients in China.

## Conclusion

In summation, our investigation unveils the complex tapestry of factors shaping online health information-seeking behaviors among gynecologic oncology patients in China. The insights gleaned from this study lay a solid groundwork for devising targeted patient education and communication strategies, ultimately fostering enhanced patient engagement and healthcare outcomes in the burgeoning digital age. Through a nuanced understanding of the demographic and psychological factors at play, healthcare providers can better navigate the digital health information landscape alongside their patients, driving forward the agenda of informed patient-centric care in the digital epoch.

## Data availability statement

The original contributions presented in the study are included in the article/Supplementary material, further inquiries can be directed to the corresponding author.

## Ethics statement

This investigation was undertaken with the sanction of the Ethics Committee of The Affiliated Hospital of Southwest Medical University (Ethics code number: KY2023200). All methods were conducted in compliance with relevant guidelines, regulations, and the Declaration of Helsinki. All participants provided written informed consent before participating in the study.

## Author contributions

WX: Data curation, Investigation, Software, Writing – original draft. XL: Writing - review & editing. YH: Data curation, Writing – original draft. LH: Conceptualization, Data curation, Writing – original draft.
