# Supplementary material for: Predicting online information seeking on Douyin, Baidu, and other Chinese search engines among gynecologic oncology patients: a cross-sectional study
Source: Front Psychol. 2023 Nov 23;14:1255604. doi: 10.3389/fpsyg.2023.1255604 (PMC10702208; doi:10.3389/fpsyg.2023.1255604)
Supplement: Supplementary file 1 [file Data_Sheet_1.docx]

**Questionnaire on Health Information-Seeking Behaviors Among Gynecologic Oncology Patients**

**Part A: Demographic Information**

1. **Age:** _______ years
2. **Marital Status:**
   - Single
   - Married/Partnered
   - Divorced
   - Widowed
3. **Highest Level of Education Completed:**
   - Less than high school
   - High school or equivalent
   - Technical school/some college
   - College degree (3- or 4-year)
   - Graduate/professional degree
4. **Annual Income:**
   - Below 25,000
   - 25,000 - 49,000
   - 50,000 - 99,000
   - 100,000 or above
   - Prefer not to say

**Part B: Clinical Features**

1. **Type of Gynecologic Cancer:**
   - Cervical Cancer
   - Ovarian Cancer
   - Uterine Cancer
   - Vaginal Cancer
   - Vulvar Cancer
   - Other (please specify): ___________
2. **Histological Type (if known):**
   - Serous
   - Endometrioid
   - Mucinous
   - Clear Cell
   - Other (please specify): ___________
3. **Grading (if known):**
   - G1 (Well differentiated)
   - G2 (Moderately differentiated)
   - G3 (Poorly differentiated)
   - GX (Cannot be assessed)
4. **Stage at Diagnosis (if known):**
   - Stage I
   - Stage II
   - Stage III
   - Stage IV
   - Not Sure
5. **Have you already received treatment for your cancer?**
   - Yes
   - No
6. **If yes, please check all types of treatment you have received:**
   - Surgery
   - Radiation Therapy
   - Chemotherapy
   - Hormone Therapy
   - Targeted Therapy
   - Immunotherapy
   - Other (please specify): ___________
7. **If you have received treatment, please provide the date of your last treatment (if known):** ___________

**Part C: Psychological Well-being**

Utilizing the State-Trait Anxiety Inventory (STAI) and the Hospital Anxiety and Depression Scale (HADS)

**Part D: Online Health Information-Seeking Behavior**

1. **Do you use online platforms to seek health information regarding your diagnosis or condition?**
   - Yes
   - No
2. **If yes, please check the platforms you use:**
   - Douyin (TikTok)
   - Baidu
   - Other Chinese search engines (please specify): ___________
   - Other platforms (please specify): ___________
3. **What type of information do you usually look for? (You may select more than one)**
   - Diagnosis and symptoms
   - Treatment options
   - Side effects of treatments
   - Support groups or forums
   - Other (please specify): ___________
4. **How often do you look for health information online?**
   - Daily
   - Weekly
   - Monthly
   - Occasionally
   - Never

**Thank you for participating in this survey. Your input is invaluable in understanding the health information-seeking behaviors among gynecologic oncology patients.**
