# Supplementary material for: Predicting online information seeking on Douyin, Baidu, and other Chinese search engines among gynecologic oncology patients: a cross-sectional study
Source: Front Psychol. 2023 Nov 23;14:1255604. doi: 10.3389/fpsyg.2023.1255604 (PMC10702208; doi:10.3389/fpsyg.2023.1255604)
Supplement: Supplementary file 2 [file Table_1.docx]

**Supplementary Table 1:** Clinical Characteristics of Gynecologic Oncology Patients Seeking Information on Douyin, Baidu, and Other Chinese Search Engines

| **Clinical Features** | **Yes**  (n=91) | **No**  (n=108) | **P value** |
| --- | --- | --- | --- |
| type of cancer, n (%) |  |  |  |
| Ovarian | 35 (38.5%) | 42 (38.9%) | 0.912 |
| Uterine | 26 (28.6%) | 33 (30.6%) | 0.776 |
| Cervical | 30 (33%) | 33 (30.6%) | 0.821 |
| histology, n (%) |  |  |  |
| Serous | 20 (22%) | 24 (22.2%) | 0.955 |
| Endometrioid | 19 (20.9%) | 22 (20.4%) | 0.920 |
| Clear Cell | 18 (19.8%) | 20 (18.5%) | 0.872 |
| Mucinous | 17 (18.7%) | 19 (17.6%) | 0.848 |
| grading, n (%) |  |  |  |
| G1 | 30 (33%) | 35 (32.4%) | 0.931 |
| G2 | 31 (34.1%) | 36 (33.3%) | 0.890 |
| G3 | 30 (33%) | 37 (34.3%) | 0.873 |
| stage at diagnosis, n (%) |  |  |  |
| I | 22 (24.2%) | 26 (24.1%) | 0.988 |
| II | 23 (25.3%) | 27 (25%) | 0.961 |
| III | 24 (26.4%) | 28 (25.9%) | 0.942 |
| IV | 22 (24.2%) | 27 (25%) | 0.920 |
| if already treated, n (%) |  |  |  |
| Yes | 45 (49.5%) | 54 (50%) | 0.966 |
| No | 46 (50.5%) | 54 (50%) | 0.966 |
| treatment type, n (%) |  |  |  |
| Surgery | 30 (33%) | 35 (32.4%) | 0.920 |
| Radiation | 31 (34.1%) | 36 (33.3%) | 0.894 |
| Chemotherapy | 30 (33%) | 37 (34.3%) | 0.876 |

**Notes:** The table provides an overview of the clinical characteristics of gynecologic oncology patients, categorized based on their information-seeking behavior (Yes or No) on Douyin, Baidu, and other Chinese search engines. Clinical features including type of cancer, histology, grading, stage at diagnosis, treatment status, and type of treatment are presented. The data are shown as the number (n) and percentage (%) of patients within each category. The P value column denotes the statistical significance of the differences between the two groups (Yes and No) for each clinical characteristic, with a P value less than 0.05 considered as statistically significant.
